# Associations of ambient air pollutants with regional pulmonary tuberculosis incidence in the central Chinese province of Hubei: a Bayesian spatial-temporal analysis

**DOI:** 10.1186/s12940-020-00604-y

**Published:** 2020-05-14

**Authors:** Fuqiang Liu, Zhixia Zhang, Hongying Chen, Shaofa Nie

**Affiliations:** 1 Department of Epidemiology and Biostatistics, School of Public Health, Tongji Medical College, Huazhong University of Science and Technology, Wuhan, 430000 Hubei People’s Republic of China; 2 Wuchang University of Technology, Wuhan, 430000 Hubei People’s Republic of China; 3 Hubei Centre for Disease Prevention and Control, Wuhan, 430000 Hubei People’s Republic of China

**Keywords:** Pulmonary tuberculosis, Air pollutants, Spatial-temporal analysis

## Abstract

**Background:**

Air pollution and pulmonary tuberculosis (PTB) are still serious worldwide problems, especially in areas of developing countries. Whether there is an association between high ambient air pollutant concentrations and PTB has not been fully explored.

**Methods:**

Bayesian spatial-temporal models were constructed to analyse the association between ambient air pollutants (particulate matter with aerodynamic diameters of ≤10 μm (PM_10_), sulfur dioxide (SO_2_) and nitrogen dioxide (NO_2_)) and PTB incidence, adjusting for socioeconomic covariates. We collected data on pulmonary TB, ambient air pollution (PM_10_, SO_2_ and NO_2_) concentrations and socioeconomic covariates from 17 prefectures in the central Chinese province of Hubei between Jan 1, 2006, and Dec 31, 2015.

**Results:**

For every annual 10 μg/m^3^ increase in SO_2_, the relative risk (RR) of PTB incidence was 1.046 (95% credible interval [CI], 1.038–1.054) in the study area. Moreover, we found positive associations with each annual 10 μg/m^3^ increase in ambient air pollutants (PM_10_, SO_2_ and NO_2_) in females but only with SO_2_ in males. A significant association for each 10 μg/m^3^ increase in SO_2_ was observed in all the age groups, with a significant association for PM_10_ only in children under 14 years of age. A significant response relationship was also observed at a 0–1 month moving average lag for each 10 μg/m^3^ increase in SO_2_.

**Conclusions:**

High ambient air pollution concentrations in areas of developing countries might increase the risk of regional PTB incidence, especially for women and young people. Precautions and protective measures and efforts to reduce ambient air pollutant concentrations should be strengthened in developing countries.

## Introduction

Although tuberculosis (TB) is likely to have emerged approximately 70,000 years ago, it remains a major health problem worldwide [1, 2]. In the past two centuries, almost one billion people have been killed by this infectious disease, with the top cause of death from a single infectious disease worldwide as human immunodeficiency virus (HIV) [3]. Currently, TB patients are mainly concentrated in developing areas, such as Southeast Asia, the Western Pacific (58%) and Africa (27%) [4]. Of the estimated 9.6 million people who developed tuberculosis globally in 2014, the top three countries with infections were India, Indonesia and China, accounting for 23, 10 and 10% respectively [4]. According to information recently published by the World Health Organization (WHO), the 20 countries with the highest TB burden based on the absolute number of incident cases and 10 countries with the highest TB burden based on the severity of the disease are all developing countries [5]. As a developing country with a high TB burden, China is currently facing a serious health problem due to the prevalence of tuberculosis [5, 6]. Despite a decrease in smear-positive TB cases, from 170 to 59 cases per 100,000 individuals during 1990–2010, TB has always ranked among the top five on the national list of notifiable infectious diseases in China [7]. Since TB can lead to substantial chronic lung disability, the loss of working ability and death among people in the most economically productive age groups [8], high TB incidences in developing countries will further aggravate the disease burden among low-income populations and hinder social and economic development.

Approximately one-third of the world’s population is estimated to have been infected with *Mycobacterium tuberculosis*, and 5–10% of those infected may ultimately develop TB [9]. However, weakened immune systems may cause TB infection to reactivate [10]. Tumour necrosis factor (TNF)-α and interferon (IFN-γ) play a crucial role in inhibiting the growth of mycobacteria [11]. An experiment was conducted with animals to examine the effect of diesel exhaust particles on mycobacterial infection and found a decrease in the levels of TNF-α and IFN-γ [12]. Some ecological studies have also suggested that TB is associated with air pollution. In the USA, researchers found potential associations between long-term exposure to particulate matter and PTB disease in North Carolina residents during 1993–2007 [13]. They also observed positive associations between PTB and NO_2_ in a nested case-control study in northern California [14]. In South Korea, the interquartile increase in SO_2_ concentration was associated with a 7% increase in TB incidence in males [15]. In China, it has also been suggested that air quality is related to TB prevalence based on a geographically weighted regression model for the exploration of ecological factors [16].

All the regions of the world are affected by air pollution, but people in low-income countries are the most impacted. According to the latest global urban air pollution database from the WHO, 98% of cities in low- and middle-income countries with more than 100,000 inhabitants that monitored air pollution exceeded the WHO limits during 2008–2015 [9]. The levels of urban air pollution in low- and middle-income countries in the eastern Mediterranean and southeast Asia were highest in the world, with values five to ten times more than the WHO guidelines [17]. In more than two-thirds of the cities in these countries, air pollution levels increased more than 5% during the five-year period from 2008 to 2013. Due to the development of industrialization and rapidly growing number of transportation vehicles in China, air pollution caused by emissions of multiple pollutants and vehicle exhaust has become a major problem, threatening public health [18, 19]. To improve air quality levels, China has taken a series of actions to maintain the air pollution emission load within a permissible range, but the situation of air pollution with PM_10_, SO_2_, and NO_2_ is still very serious [20, 21].

Since the levels of air pollution and PTB incidence are high in developing countries, if air pollution is indeed associated with the rates of TB disease, then high air pollution concentrations in developing countries will impact efforts of TB control. There is not a clear understanding of whether high concentrations of certain (or if any) air pollutants are impacting the risk of PTB incidence in developing countries. Moreover, there are variable gender and age-related differences in the effects of air pollution on PTB. On the other hand, data related to air pollution and notifiable PTB cases are often collected over space and time. The association between air pollutants and PTB incidences may vary spatiotemporally, and the relation can differ depending on location (in the spatial domain) or temporal patterns (in the spatial-temporal domain).

However, most previous studies used traditional regression models to determine the relationships between PTB disease and air pollution, which do not account for any residual spatial-temporal variation in the outcome that is not already captured by the exposures [13–15, 22–24]. Bayesian models, which have been widely applied in recent years, can fully utilize the temporal and spatial information of data and prior knowledge [25, 26]. In this study, using data from the central Chinese province of Hubei for 2006 to 2015, we aimed to assess the impact of air pollutants (SO_2_, NO_2_ and PM_10_; the only three air pollutants monitored by the Department of Hubei Environmental Protection before 2012) on PTB incidence by Bayesian approaches that included spatial and temporal random effects.

## Methods

### Study area

Hubei Province is located between longitudes 108°21′ and 116°07′ east and latitudes 29°05′ and 33°20′ north in Central China, with a total area of 185,900 km^2^ and a population of 58.16 million in 2015. Hubei Province is divided into 17 prefectures: 1 sub-provincial level city, 11 prefecture-level cities, 4 prefectures and 1 autonomous prefecture.

### PTB incidence

Data, including the demographic information (e.g., gender, age, and address) from PTB case notifications, were available from the Hubei Centre for Disease Prevention and Control (CDC) through the Chinese information system for disease control and prevention. PTB cases were diagnosed using X-ray, pathogen detection, and pathological diagnosis, according to the diagnosis criteria recommended by the National Health and Family Planning Commission of the People’s Republic of China for the period from Jan 1, 2006, to Dec 31, 2015. Annual and monthly numbers of pulmonary TB case notifications in each city were retrieved from the system. PTB incidences were calculated as the number of PTB cases divided by the annual average population. Since the PTB data were secondary and properly anonymized and informed consent was obtained by the CDC at the time of the original data collection, ethical approval was not required.

### Air pollutant exposure

Measurements of ambient air pollutant concentrations in 17 prefectures were obtained from the Hubei Environmental Air Monitoring Networks, which consisted of monitoring stations with the numbers from 17 in 2006 to 58 in 2015, scattered in each city and maintained by the Department of Hubei Environmental Protection. Each station provided daily concentrations of PM_10_, NO_2_, and SO_2_ from 24-h continuous monitoring. The monitoring behaviour and data quality were evaluated and supervised each year, according to the Hubei Environmental Monitoring Quality Control Assessment Program. In the present study, for PM_10_, NO_2_, and SO_2_, air pollutant exposure was defined as the yearly and monthly average of the 24-h measurements over all the monitors within the 17 cities in the entire study period.

### Socioeconomic covariates

The socioeconomic covariates that were considered potential confounding factors in our analyses included the population density, proportion of the elderly population, per capita disposable income, per capita housing area, per capita Engel coefficient, per capita tobacco consumption, number of health technicians per thousand individuals, average temperature and humidity, and HIV incidence at the city level. The data were obtained from a Hubei population-based information sharing platform, Hubei Statistical Yearbook, the Hubei Tobacco Monopoly Bureau, and the Chinese information system for disease control and prevention.

### Statistical analyses

Bayesian approaches use information from samples and prior distributions to estimate posterior distribution parameters, which can be done by using a Markov chain Monte Carlo (MCMC). Here, denote y_*it*_ and n_*it*_ as the number of TB cases and the population in city *i* at time *t*, respectively, for *i* = 1, …, m cities and t = 1, …, *T* years or months. Then, y_*it*_ ~ Poisson (E_*it*_θ_*it*_), E_*it*_ = n_*it*_Σ_it_ y_*it*_/Σ_it_ n_*it*_, where E_*it*_ is the expected number of TB cases and θ_*it*_ is the relative risk (RR) of PTB in city *i* at time *t*. The Bayesian spatial-temporal regression models were constructed by a Poisson model with a log link, and the best fitting model was selected to quantify the associations between air pollutants and pulmonary TB incidence. Accounting for collinearity or potential interactions between the variables, all the socioeconomic covariates were entered into the linear regression model as potential confounders and were screened with backwards elimination and a 10% change-in-estimate criterion by the stepwise method. The covariates were then entered into the Bayesian models for further analysis.

We constructed four Bayesian models with socioeconomic covariates. The first Bayesian model was a non-spatial model, built as: log (θ_*it*_) = *β*_0_ + *β*_*k*_*X*_*it*_ + μ_i_. In this model, *X*_*it*_ is the independent variable in city *i* at time *t* and includes the concentrations of ambient air pollutants and socioeconomic covariates. *β*_0_ represents the intercept of all the cities and times with a flat distribution (*β*_0_ ~ dflat()), *β*_*k*_ represents the coefficients of the parameters, assigned as non-informative normal priors (*β*_*k*_ ~ normal (0, 100)), and μ_i_ is a non-spatially structured random effect caused by other non-spatial factors, following a normally distributed prior with a zero mean and precision equal to 0.001. The second model was a spatial model, which considered non-spatial and spatial random effects: log (θ_*it*_) = *β*_0_ + *β*_*k*_*X*_*it*_ + *μ*_*i*_ + *ν*_*i*_. In this model, *ν*_*i*_ is a random effect that is spatially structured by prefecture to account for spatial autocorrelation, which was assumed to be a conditionally autoregressive (CAR) prior, in which the weights were derived from an adjacency-based first-order spatial proximity matrix. A uniform prior was specified for *ν*_*i*_ (*ν*_*i*_ ~ normal (0, σ_*v*_^2^)), where σ_*v*_^2^ is the variance of *ν*_*i*_. The third model was a spatiotemporal model, which included nonspatial, spatial and temporal random effects: log (θ_*it*_) = *β*_0_ + *β*_*k*_*X*_*it*_ + μ_i_ + *ν*_*i*_ + *g*_*t*_*.* Here, *g*_*t*_ is explained as the temporal variations, considering the spatial effects to be independent at different times, following an autoregressive (AR) prior with a normal distribution (*g*_*t*_ ~ normal (0, σ_*g*_^2^)), where σ_*g*_^2^ is the variance of *g*_*t*_. The fourth model was a spatiotemporal interactive model, in which the spatiotemporal interactive effects were added to the spatiotemporal model, resulting in the form log (θ_*it*_) = *β*_0_ + *β*_*k*_*X*_*it*_ + μ_i_ + *ν*_*i*_ + *g*_*t*_ *+ psi*_*it*_*.* In this equation, *psi*_*it*_ is a spatial-temporal interaction effect, assuming random spatial effects in each time as a conditionally autoregressive process, with the non-informative prior *psi*_*it*_ ~ normal (0, 1000). The model with the smallest deviance information criterion (DIC) value was considered the most appropriate model. Additionally, to explore the lag response relationship of ambient air pollutants on regional TB incidence, we also analysed the lag elapses in various months by the Bayesian spatial-temporal model. All the analyses were conducted with SPSS statistics 19.0.0 and WinBUGS 1.4.3 software.

## Results

**Fig. 1:**
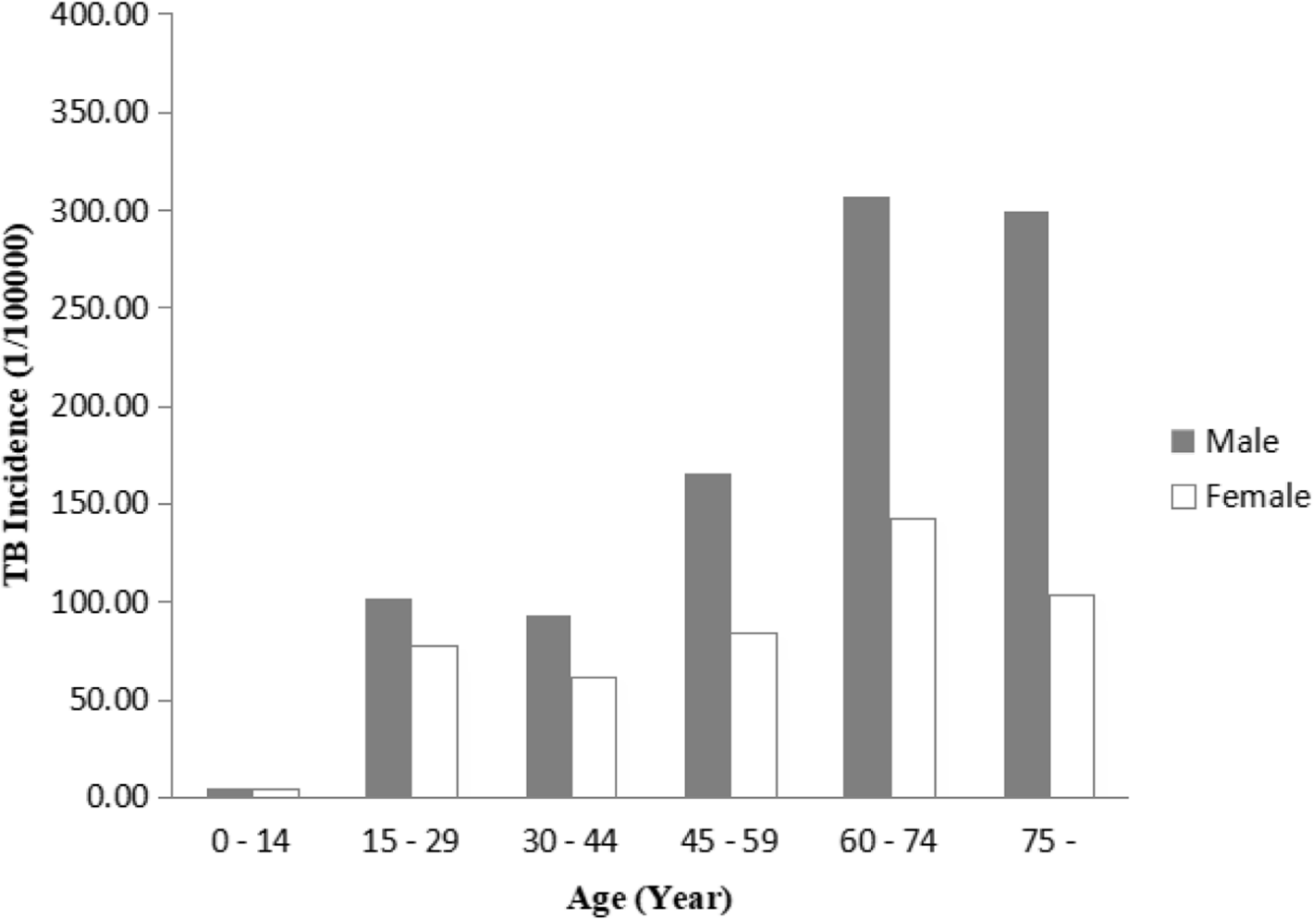
Total PTB incidence by gender and age in Hubei Province, China

**Fig. 2:**
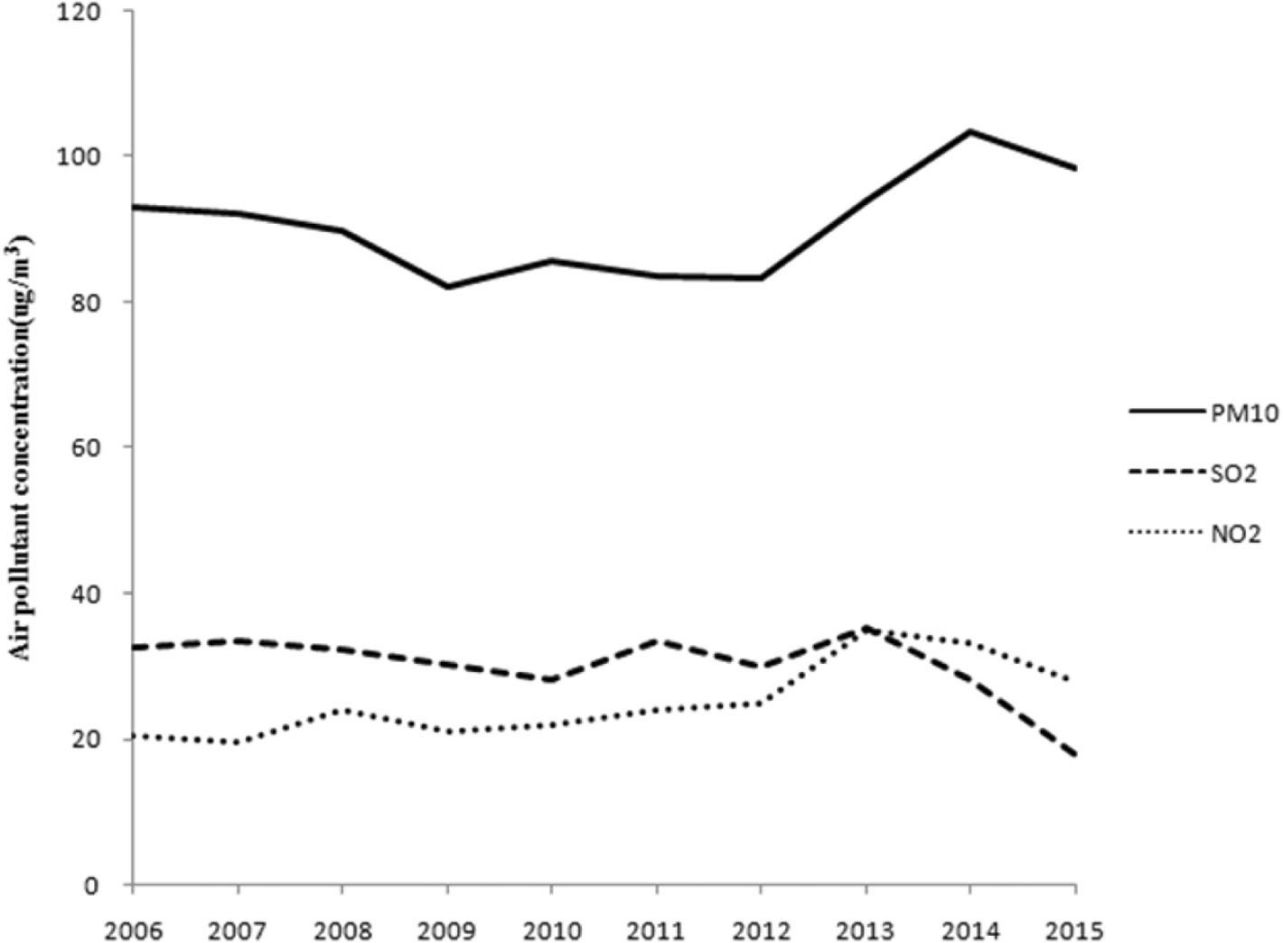
Trend of annual air pollutant concentrations in Hubei Province, 2006–2015

**Fig. 3:**
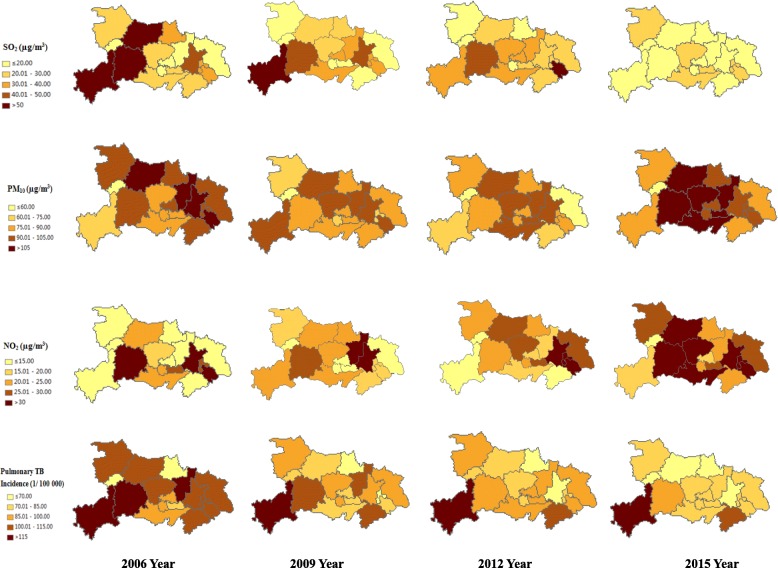
Spatial distributions of air pollutants and pulmonary TB incidence in Hubei Province, 2006–2015

During the study period, there were 91.83 total average PTB case notifications per 100,000 individuals, decreasing from 108.23 per 100,000 individuals in 2006 to 78.14 per 100,000 individuals in 2015 (Table 1). The risk of PTB increased with age, with the lowest PTB incidence in children under 14 years old. From a gender perspective, rates of reported PTB incidence were lower for women in all the age groups, with an average male-to-female sex ratio of 2.08 (Fig. 1). The total average concentrations of the air pollutants (PM_10_, SO_2_ and NO_2_) were 90.7, 30.4 and 25.2 μg/m^3^, respectively (Table 2). Of these, the annual averages of PM_10_ were higher than the current Ambient Air Quality Standard of China [27]. A moderate rise in the annual concentrations of PM_10_ and NO_2_ was found; the mean values of PM_10_ increased from 93 in 2006 to 99 μg/m^3^ in 2015, and the mean values of NO_2_ increased from 20 in 2006 to 28 μg/m^3^ in 2015. There was a relatively steady decline in the annual concentrations of SO_2_, from 33 in 2006 to 18 μg/m^3^ in 2015 (Fig. 2). To visualize the spatial-temporal trends, we plotted the spatial distributions of the air pollutants and PTB incidence among the 17 prefectures in Hubei Province (Fig. 3).

**Table 1:** Demographic characteristic of PTB incidence (per 100,000 population) in Hubei Province, 2006–2015

	2006	2007	2008	2009	2010	2011	2012	2013	2014	2015	Total
Age											
0- year	6.50	5.30	4.42	3.84	3.56	4.13	4.63	4.01	4.05	4.41	4.52
15- year	115.84	112.35	106.83	91.12	88.62	89.32	89.43	84.02	78.18	71.21	93.09
30- year	108.62	103.83	95.12	78.79	73.89	71.88	73.69	70.87	67.77	64.27	81.88
45- year	179.63	186.59	173.28	131.11	120.07	122.80	111.89	103.90	99.71	98.29	128.78
60- year	326.75	339.31	305.74	235.42	213.81	199.83	200.51	188.56	184.75	190.92	231.90
75- year	234.92	281.80	293.64	219.31	192.80	179.88	166.51	158.47	146.27	150.27	192.70
Sex											
Male	142.28	148.18	141.33	120.28	113.61	118.75	119.24	112.53	107.72	106.14	122.96
Female	72.60	73.54	70.89	58.62	53.41	55.71	54.29	52.21	50.11	49.11	58.98
Total	108.23	112.43	107.55	90.70	84.75	88.11	87.67	83.14	79.58	78.14	91.83

**Table 2:** Annual ambient air pollutant concentrations of 17 cities in Hubei Province, China, 2006–2015

	Mean	SD	Min	5%	Median	95%	Max	IQR
PM_10_(μg/m^3^)	90.7	20.0	31.0	44.1	93.5	117.8	150.0	24.0
SO_2_(μg/m^3^)	30.4	15.3	8.0	11.6	29.0	56.9	95.0	19.0
NO_2_(μg/m^3^)	25.2	12.1	3.0	9.0	23.0	54.0	60.0	16.0

Ambient air quality standards set by Ministry of Ecology and Environment of the people’s Republic of China [27]

PM_10_ ≤ 70 μg/m^3^ (annual), NO_2_ ≤ 40 μg/m^3^ (annual), SO_2_ ≤ 60 μg/m^3^ (annual)

PM_10_, particulate matter with aerodynamic diameter of ≤10 μm; SO_2_, sulfur dioxide; NO_2_, nitrogen dioxide; SD, standard deviation; Min, minimum; Max, maximum; 5%, IQR, interquartile rang

Table 3 shows that the deviance information criterion (DIC) value was lowest in the spatial-temporal interactive model among the four Bayesian models. Therefore, the spatial-temporal interactive model was used for further analysis.

**Table 3:** The deviance information criterion (DIC) for Bayesian models

Bayesian Model	DIC
Non-spatial model	22,983.2
Spatial model	22,144.5
Spatial-temporal model	19,501.5
Spatial-temporal interactive model	1887.6

Table 4 shows the estimated RR and 95% confidence intervals (CIs) based on the spatial-temporal interactive model, adjusting for socioeconomic covariates. Every 10 μg/m3 increase in SO_2_ concentration increases the risk of regional PTB incidence by 4.6% (RR = 1.046; 95% CI, 1.038–1.054). However, PM_10_ and NO_2_ were not significantly associated with regional PTB case notifications. When the model analysis was stratified by gender, positive associations were observed among females, with a 1.0, 2.0 and 1.5% increase in the risk of regional PTB for every 10 μg/m^3^ increase in PM_10,_ SO_2_ and NO_2,_ respectively. Among males, positive associations were found only for SO_2_, with a 5.4% increase in the risk of PTB for each 10 μg/m^3^ increase. To test whether associations of air pollutants and regional PTB differed by age, the analysis was also stratified into three age groups (< 15, 15–59 and ≥ 60 years). The results showed that PM_10_ was positively associated with regional PTB only in children under 15 years of age (RR = 1.018; 95% CI, 1.007–1.030). Positive associations were also observed for SO_2_, with a 2.2, 6.4 and 1.8% increase in the PTB risk of individuals < 15, 15–59 and ≥ 60 years old, respectively. However, the observed increased risk was not statistically significant for NO_2_.

**Table 4:** Every 10 μg/m^3^ increase in ambient air pollutant concentration on PTB incidence by spatial-temporal interactive model

	PM_10_		SO_2_		NO_2_	
	RR	95% CI	RR	95% CI	RR	95% CI
All	1.002	0.988–1.019	1.046	1.038–1.054	1.008	0.986–1.028
Stratified by gender						
Male	0.994	0.971–1.017	1.054	1.045–1.063	0.998	0.981–1.016
Female	1.010	1.002–1.017	1.020	1.017–1.023	1.015	1.006–1.025
Stratified by age						
< 15 years	1.018	1.007–1.030	1.022	1.008–1.036	0.998	0.979–1.019
15–59 years	0.988	0.964–1.011	1.064	1.050–1.078	1.010	0.989–1.022
≥ 60 years	1.006	0.993–1.035	1.018	1.010–1.027	1.005	0.980–1.031

Abbreviations: *CI* Credible interval; *PTB* Pulmonary tuberculosis; *PM*_*10*_ Particulate matter with aerodynamic diameter of ≤10 μm; *SO*_*2*_ Sulfur dioxide; *NO*_*2*_ Nitrogen dioxide; *RR* Relative risk

**Fig. 4:**
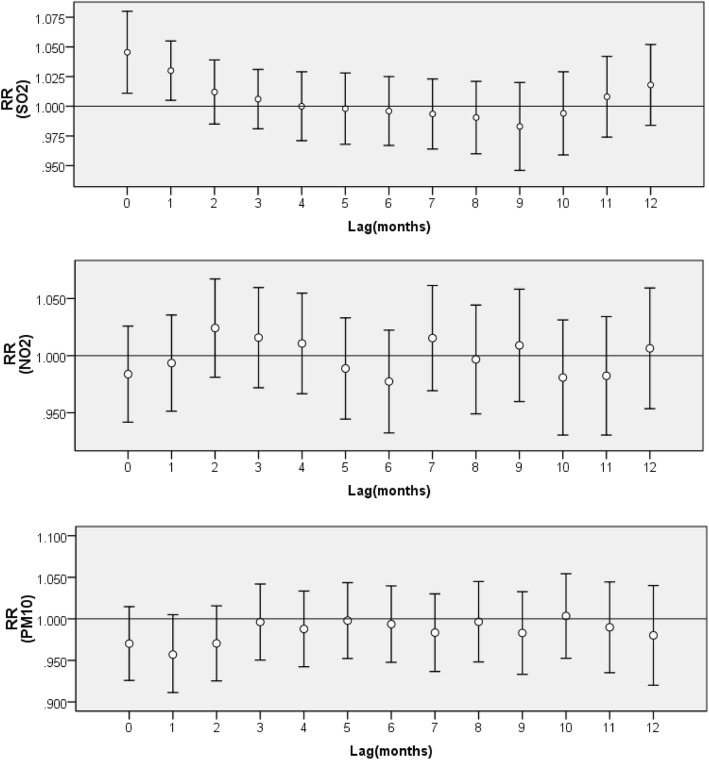
The RRs and 95% CIs of a 10 μg/m3 increase in ambient air pollutants on PTB at different lag months by spatial-temporal interactive model in Hubei Province. RR, relative risk; CI, credible interval; PTB, pulmonary tuberculosis; PM_10_, particulate matter with aerodynamic diameter of ≤10 μm; SO_2_, sulfur dioxide; NO_2_, nitrogen dioxide; IRR, incidence rate ratio

Figure 4 shows the lag associations between the ambient air pollutant concentrations and regional PTB based on the spatial-temporal interactive model, adjusting for socioeconomic covariates. We observed a significant response relationship, with a 0–1 month moving average lag for each 10 μg/m^3^ increase in SO_2_. However, no significant associations were observed in PM_10_ and NO_2_.

## Discussion

Previously, the relationships between air pollutants and TB were explored by many traditional methods, including linear models, conditional logistic regression models, Poisson regression models and hierarchical Bayesian methods [14–16, 21, 28]. However, few studies have considered spatial or spatial-temporal interactive effects at the population level. Considering the goodness of fit based on the DIC value, we adopted a Bayesian spatial-temporal interactive model in our study. This model could identify spatial differences and solve problems such as spatial autocorrelation, which is difficult for most traditional statistical methods. Hubei Province is one of the most serious tuberculosis epidemic areas in China. The rates of PTB incidence as well as the annual air pollutant concentrations of PM_10_, SO_2_ and NO_2_ in our study are higher than those in developed countries [4, 20, 21]. Our findings showed that SO_2_ was positively associated with PTB incidence, with a 4.6% increase in PTB incidence rates per 10 μg/m^3^ increase in SO_2_. When stratified by gender, positive associations were noted with exposure to all three air pollutants in females but only to SO_2_ in males. When stratified by age, positive associations were observed for SO_2_ in all the age groups and for PM_10_ only in children under 15 years. Moreover, a significant lag response relationship was also found, with a lag of 0–1 month for SO_2_.

In our study, the risk of PTB was not significantly associated with exposure to PM_10_. We noticed that in previous experimental studies, exposure to PM_10_ was found to enhance intracellular *Mycobacterium tuberculosis* growth by inducing senescence and downregulating the expression of the antimicrobial peptides human β-defensin2 (HBD-2) and HBD-3, which are important in the early control of TB infection [29, 30]. However, experimental studies cannot reflect the actual association between ambient PM_10_ and TB incidence at the population level. Although one previous epidemiological study in North Carolina found that exposure to particulate air pollution increased the risk of TB during 1993–2007 [13], the study was based on Poisson regression models with low ambient PM_10_ levels (19.39–24.63 μg/m^3^) as well as a low rate of PTB disease incidence (4.41 per 100,000 persons/year); in contrast, in our research, there were high levels of air pollutant concentrations and PTB incidence. However, most recent epidemiologic studies have found no significant associations between PM_10_ and TB, which is consistent with our results [13–15]. To elucidate the possible associations between PM_10_ and PTB, more research needs to be done in the future.

The associations observed for SO_2_ were significantly positive, similar to the findings of other studies. Shilova and Glumnaia found that atmospheric pollutants (including SO_2_) were significantly associated with TB incidence in Russia [31]. Hwang et al. reported that the interquartile increase in the SO_2_ concentration in outdoor air pollutants could result in a 7% increase in TB incidence in South Korea [15]. The reason for this association may be attributed to the effect of exposure to SO_2_ on pulmonary defences. A previous study showed that a 30-min exposure to 12.5 ppm SO_2_ induced 62% death of alveolar macrophages and led to a decrease of 63% in the release of reactive oxygen species, which are crucial for inhibiting or killing *Mycobacteria tuberculosis* [32]. The researchers also found that exposure to SO_2_ caused a significant decrease in the production or release of TNF-α and interleukin-1, which can defend against *Mycobacterium tuberculosis* by regulating the activity of other cytokines and chemokines in early TB infections [27, 33]. Using TNF-neutralizing therapies increases the risk of developing tuberculosis and induces frequent reactivation of latent TB in patients [34–36]. However, compared with other studies, the effect size of SO_2_ in our study seems very small. For example, the interquartile range (IQR = 0.3 ppb) increase in SO_2_ concentration was associated with a 7% increase in TB incidence rate in South Korea, but only a 4.6% increase in TB incidence rates per 10 μg/m^3^ increase in SO_2_ concentration was observed in our study. There may be several reasons for these findings. First, the average SO_2_ concentrations and IQRs in our study were higher than those in other studies, which caused a smaller variation with the same scale change in concentrations. Second, a higher TB incidence may result in a smaller change in the TB incidence rate ratio if the effect caused the same change size in TB incidence. Third, some potential confounders, which influenced regional TB epidemiology [37], may disturb the effect of SO_2_, and no adjustments were made for this disturbance in the previous study.

No association was observed between NO_2_ and PTB, consistent with the findings of some studies. After exploring the impact of outdoor air pollution on TB in South Korea, researchers found that the concentrations of ambient NO_2_ were not associated with TB incidence^15^. Another study also showed no significant association between the daily initial TB outpatient visits and daily average concentration of NO_2_ [38]. However, a recent nested case–control study in northern California found a positive association between TB and NO_2_ [14]. One thing to note is that the study in northern California assessed average individual-level concentrations of NO_2_ for only 2 years before the diagnosis of TB; the individual-level exposure depending on only outdoor concentrations may be altered by smoking or using gas appliances indoors.

To investigate the sex-specific associations between air pollution and PTB, the analysis was stratified by gender. Positive relationships were observed for PM_10_ and NO_2_ in females but not in males. This result suggested that the effects of PM_10_ and NO_2_ on PTB may differ by gender. Other epidemiological studies also found that the effects of air pollutants on respiratory health are much more marked in females [39]. Clougherty et al. also found that increased impact of air pollution on respiratory health in females was linked to their social or behavioural and biological differences [40]. The reason may be that some sex-linked traits impact the biological transport of environmental chemicals, while gender-linked activities (i.e., where and what people spend time doing) determine the distribution of air pollution exposure. The biological sex of females leads to the inhalation of more doses of air pollution, greater deposition and absorption of air pollution and higher gas–blood barrier permeability in the respiratory tract [41, 42]. Another important reason for the sex-specific associations between air pollution and PTB may be related to a prominent feature of smoking habits in China and the substantial male/female difference in the rates of smoking. For example, a recent nationally representative survey showed that the male/female ratio of smoking was 22 in 2010 [43]. The increased relative risk degree of PTB incidence by air pollution exposure may be obscured or weaken partially by heavy smoking in men. Further stratifying the analysis by age, we observed positive associations for PM_10_ in individuals aged 0–14 years but not in those aged 15 years or older. Evidence from epidemiological studies also found that the effects of air pollution on respiratory health are much more marked in children [44–46]. This may be due to the differences in breathing pattern and lung structure between children and adults. The dose of air pollutants deposited the respiratory systems of children is higher than that of adults if they are exposed to the same levels of air pollutants [47, 48]. By spending more time on activities outdoors, children also increased their ventilation rates and exposure to air pollutants [49]. Therefore, we supposed that the gender- and age-related effects of air pollutants on pulmonary TB in our study may be due to some interplay between the above mentioned factors.

As with any statistical modelling study, our study also has some limitations. First, despite the biologic plausibility of a possible association between air pollutants and PTB, our results should be interpreted cautiously, with the inherent limitations of an ecological study design that used population-level data. Second, some key potential confounders to the relationship between air pollution and regional PTB, including household air pollution from the burning of solid fuel and more detailed information on smoking, should be accounted for in future investigations, although city-level tobacco consumption data were collected. Third, exposure to ambient PM_2.5_ is arguably an equally relevant indicator of air pollution exposure, but is not included given the unavailability of PM_2.5_ estimates from the Hubei monitoring network before 2012. Last but not least, due to the implementation of the Chinese TB control project and policy shifts, there are also potential issues with the identification of TB, including changes in active TB screening, access to or use of TB treatment, case registration report rate, and others. Future epidemiological cohort studies are needed for the assessment of cause-specific TB disease, especially in females and young people.

## Conclusions

This study shows that long-term exposure to high ambient air pollutant concentrations in areas of developing countries increased the risk of regional PTB, especially for women and young people. Precautions and protective measures and efforts to reduce ambient air pollutant concentrations should be strengthened in developing countries.

## Data Availability

The data can be accessed from the Hubei CDC and Department of Hubei Environmental Protection with permission via direct request.

## Abbreviations

CDC: Centre for disease prevention and control
CI: Credible interval
DIC: Deviance information criterion
HIV: Human immunodeficiency virus
RR: Relative risk
NO_2_: Nitrogen dioxide
PM_10_: Particulate matter with aerodynamic diameter of ≤10 μm
PTB: Pulmonary tuberculosis
SO_2_: Sulfur dioxide
TB: Tuberculosis
